# Supplementary material for: Biogeographic evidence supports the Old Amazon hypothesis for the formation of the Amazon fluvial system
Source: PeerJ. 2021 Nov 25;9:e12533. doi: 10.7717/peerj.12533 (PMC8627654; doi:10.7717/peerj.12533)
Supplement: Supplemental Information 1 [file peerj-09-12533-s001.doc]

**Supplemental files**

**Data S1.** GenBank accession numbers of genes included for each phylogeny.

**Table S1.** Digital Object Identifier (DOI) from GBIF occurrences.

**Table S2.** List of fossils used in the present study.

**Table S3.** Bayesian Factors for each gene of all ingroups.

**Table S4.** Stratified models for each phylogeny.

**Figure S1.** Areas used in the analyses: G= Guaina, I= Imeri, P=Napo, N=Marañon, J=Ucayali, U=Jurua, A=Purus, R=Rondonia, T=Tapajos, X=Xingu, C=Araguaia, B=Belem.

**Figure S2.** Lineage through-time for all taxa.

**Figura S3**. Methodology workflow.

**Supplementary references**

**Data S1.**

**Rhinella**

| **Specie** | **Cytb** | **Rhod** | **16s** | **12s-trna-val-16s** | **Rag1** | **Pomc** |
| --- | --- | --- | --- | --- | --- | --- |
| *Anaxyrus_americanus* | AB159264 | NA | KF665122 | DQ158426 | KJ609650 | DQ158268 |
| *Anaxyrus_woodhousii* | AY288067 | NA | NA | DQ158498 | KJ609653 | DQ158339 |
| *Anaxyrus_cognatus* | NA | NA | NA | DQ158444 | KJ609656 | DQ158285 |
| *Anaxyrus_exsul* | NA | NA | NA | AY325990 | DQ158372 | DQ158291 |
| *Cranopsis_coccifer* | HM563944 | NA | AY927856 | DQ158443 | KJ609669 | DQ158284 |
| *Cranopsis_coniferus* | HM563945 | NA | FJ784601 | DQ158445 | HM563988 | DQ158286 |
| *Cranopsis_valliceps* | AY008230 | NA | AY008229 | DQ158493 | KJ609665 | DQ158334 |
| *Cranopsis_alvarius* | HM563933 | NA | HM563860 | DQ158425 | KJ609663 | DQ158267 |
| *Bufo_bufo* | AB159262 | NA | AY555021 | DQ158438 | AY583336 | KT239445 |
| *Bufo_andrewsi* | AF174502 | NA | AF160782 | DQ158428 | DQ158353 | DQ158270 |
| *Amietophrynus_gracilipes* | NA | NA | NA | DQ158456 | DQ158378 | DQ158297 |
| *Amietophrynus_regularis* | NA | NA | KX671722 | DQ158485 | KJ609677 | DQ158326 |
| *Amietophrynus_maculatus* | NA | NA | NA | DQ158469 | KJ609678 | DQ158311 |
| *Duttaphrynus_melanostictus* | AY247259 | NA | NA | DQ158475 | KJ609681 | KU183158 |
| *Schismaderma_carens* | NA | NA | KF665363 | DQ158424 | KF666220 | DQ158266 |
| *Ingerophrynus_macrotis* | NA | NA | NA | DQ158468 | KF666244 | KU183150 |
| *Peltophryne_lemur* | NA | NA | U52787 | DQ158465 | DQ158386 | DQ158306 |
| *Rhaebo_haematiticus* | HM563930 | NA | KR863345 | DQ158461 | KJ609683 | DQ158302 |
| *Rhaebo_nasicus* | NA | NA | NA | DQ158477 | DQ158396 | DQ158319 |
| *Nannophryne_cophotis* | NA | NA | NA | DQ158446 | DQ158369 | DQ158287 |
| *Dendrophryniscus* | KX025647 | NA | KU495200 | AY326000 | DQ503337 | AY819081 |
| *Melanophryniscus_klappenbachi* | DQ502444 | DQ283765 | NA | AY843699 | AY844478 | KP295580 |
| *Melanophryniscus_stelzneri* | NA | NA | U52782 | DQ158421 | KF666223 | DQ158263 |
| *Rhinella_achavali* | KC567990 | HM159237 | GU178798 | GU178787 | NA | NA |
| *Rhinella_amboroensis* | NA | DQ284003 | NA | DQ283386 | NA | NA |
| *Rhinella_arenarum* | HM159228 | AY844547 | GU178796 | DQ158429 | AY844370 | DQ158271 |
| *Rhinella_arequipensis* | NA | NA | NA | DQ158430 | DQ158355 | DQ158272 |
| *Rhinella_arunco* | KC817199 | NA | NA | DQ158442 | DQ158365 | DQ158283 |
| *Rhinella_atacamensis* | KC778243 | NA | NA | DQ158433 | JX442329 | DQ158275 |
| *Rhinella_azarai* | KP684987 | KP685164 | NA | KP685186 | KP685115 | KP685079 |
| *Rhinella_bergi* | KP684990 | KP685165 | NA | KP685189 | KP685118 | KP685084 |
| *Rhinella_bernardoi* | KP684991 | KP685166 | NA | KP685194 | KP685120 | KP685086 |
| *Rhinella_castaneotica* | NA | NA | KU495498 | DQ158440 | DQ158364 | DQ158282 |
| *Rhinella_centralis* | KP684992 | KP685167 | JN021325 | KP685195 | KP685122 | KP685087 |
| *Rhinella_cerradensis* | HM159232 | HM159238 | NA | NA | NA | NA |
| *Rhinella_crucifer* | HM159226 | KC199966 | KU495502 | DQ158447 | KJ609675 | KJ532293 |
| *Rhinella_dapsilis* | NA | NA | KR012641 | DQ158448 | DQ158370 | NA |
| *Rhinella_dorbignyi* | KP684994 | KP685168 | NA | KP685199 | KP685123 | KP685090 |
| *Rhinella_fernandezae* | KP684997 | KP685169 | NA | KP685202 | KP685124 | KP685092 |
| *Rhinella_festae* | NA | NA | KR012624 | DQ158423 | DQ158349 | DQ158265 |
| *Rhinella_granulosa* | KP685002 | KP685172 | GU178800 | KP685209 | KP685129 | KP685098 |
| *Rhinella_henseli* | NA | KC199854 | NA | KP685183 | KP685113 | KP685077 |
| *Rhinella_humboldti* | NA | KP685173 | KP149488 | KP685211 | KP685131 | KP685099 |
| *Rhinella_icterica* | HM159230 | HM159240 | KU495510 | DQ158462 | NA | DQ158303 |
| *Rhinella_jimi* | HM159234 | HM159241 | GU178795 | GU178784 | NA | NA |
| *Rhinella_limensis* | NA | NA | NA | DQ158466 | NA | DQ158307 |
| *Rhinella_major* | KP685008 | KP685175 | JF790180 | KP685219 | KP685135 | KP685105 |
| *Rhinella_manu* | NA | NA | NA | NA | DQ404395 | DQ404396 |
| *Rhinella_margaritifera* | JX298409 | HM159242 | KR012628 | NA | HM563975 | AY819080 |
| *Rhinella_marina* | KR011949 | DQ283789 | KR012644 | DQ158474 | DQ158392 | DQ158316 |
| *Rhinella_merianae* | KP685010 | KP685176 | NA | KP685222 | KP685137 | KP685107 |
| *Rhinella_mirandaribeiroi* | KP685012 | KP685177 | KF723018 | KP685228 | KP685140 | KP685109 |
| *Rhinella_nesiotes* | NA | NA | NA | DQ158478 | DQ158397 | DQ158320 |
| *Rhinella_ocellata* | NA | NA | JN867572 | DQ158479 | DQ158398 | DQ158321 |
| *Rhinella_ornata* | NA | KC199963 | KU495531 | NA | NA | NA |
| *Rhinella_poeppigii* | HM159233 | HM159243 | GU178790 | DQ158481 | KJ609674 | KJ532292 |
| *Rhinella_pygmaea* | KP685013 | KP685181 | NA | KP685229 | KP685141 | KP685110 |
| *Rhinella_rubescens* | HM159229 | HM159244 | NA | DQ158486 | NA | DQ158327 |
| *Rhinella_schneideri* | HM159235 | NA | JQ627202 | DQ158480 | KJ609673 | DQ158322 |
| *Rhinella_spinulosa* | NA | DQ283775 | NA | DQ158487 | KJ609676 | DQ158328 |
| *Rhinella_sternosignata* | NA | KP685163 | NA | KP685184 | KP685144 | KP685078 |
| *Rhinella_vellardi* | NA | NA | NA | DQ158495 | DQ158411 | DQ158336 |
| *Rhinella_veraguensis* | NA | NA | NA | DQ158496 | DQ158412 | DQ158338 |
| *Rhinella_veredas* | HM159231 | HM159245 | NA | NA | NA | NA |

**Cracidae**

| **Specie** | **Nd2** | **Cytb** | **Coi** | **Nd5** | **Cltcl1** | **Eef2** | **Tfb5** | **Rho** | **Serpinb14** | **Cltc** |
| --- | --- | --- | --- | --- | --- | --- | --- | --- | --- | --- |
| *Numida_meleagris* | NA | KF833637 | NA | NA | EU302774 | EU738650 | NA | EU737246 | KC749887 | KC749599 |
| *Megapodius_layardi* | NA | KF833615 | NA | NA | KF833495 | FJ881844 | NA | NA | KC749932 | KC749598 |
| *Ptilopachus_petrosus* | DQ768289 | AM236886 | NA | KR732873 | NA | KR732890 | NA | NA | KR732792 | KR732672 |
| *Rollulus_rouloul* | KR732854 | EF571185 | NA | NA | JF497007 | EU738688 | NA | EU737280 | KC749947 | NA |
| *Tympanuchus_phasianellus* | AF230127 | AF230181 | DQ434206 | NA | KC749662 | KC749714 | NA | NA | KC749906 | KC749617 |
| *Coturnix_japonica* | NA | DQ515818 | GQ481652 | NA | KC749626 | KC749691 | NA | DQ402463 | KC749869 | KC749581 |
| *Callipepla_californica* | KR732836 | AB120131 | JN850719 | KR732862 | NA | KR732885 | NA | KR732764 | KR732783 | KR732687 |
| *Colinus_virginianus* | KR732833 | EU372675 | NA | KR732859 | KC749627 | KR732887 | NA | KR732765 | KR732785 | KR732685 |
| *Gallus_gallus* | KF792740 | EU839454 | JF498860 | NA | EU302770 | EU738569 | NA | AB496228 | NM_205152 | KC749587 |
| *Meleagris_gallopavo* | AF222556 | HQ122602 | DQ433017 | NA | KC749642 | FJ881856 | NA | NA | NM_001303190 | KC749597 |
| *Oreophasis_derbianus* | NA | AY659805 | AF165495 | AY140759 | KX345905 | KX356290 | KX356206 | KX356149 | KX356174 | KX345877 |
| *Ortalis_vetula* | KX356238 | L08384 | KX356279 | KX356262 | KX345911 | NA | KX356214 | KX356156 | KX356180 | KC749601 |
| *Ortalis_leucogastra* | NA | NA | NA | NA | KX345908 | KX356306 | KX356211 | KX356153 | KX356177 | KX345880 |
| *Ortalis_poliocephala* | KX356257 | AY659784 | KX356278 | KX356261 | KX345910 | KX356293 | KX356213 | KX356155 | KX356179 | NA |
| *Ortalis_wagleri* | KX356239 | NA | KX356280 | NA | KF833519 | KX356296 | KX356215 | NA | KX356181 | KX345883 |
| *Chamaepetes_unicolor* | KX356231 | AY659796 | JQ174405 | NA | KX345897 | NA | KX356198 | NA | KX356168 | NA |
| *Penelopina_nigra* | NA | AY354492 | AF165499 | AY140757 | KX345923 | KX356302 | KX356227 | KX356164 | KX356192 | NA |
| *Chamaepetes_goudotii* | AY140741 | AY659795 | JN801554 | AY140755 | NA | NA | KX356197 | NA | NA | NA |
| *Penelope_argyrotis* | NA | AY659803 | NA | NA | NA | NA | NA | NA | NA | NA |
| *Penelope_barbata* | KX356252 | NA | JN801892 | NA | NA | NA | NA | NA | NA | NA |
| *Penelope_montagnii* | KX356245 | AY659802 | KX356283 | KX356267 | KX345918 | KX356305 | KX356222 | KX356161 | KX356188 | KX345890 |
| *Penelope_marail* | KX356244 | KX356277 | JQ175713 | KX356266 | KX345917 | NA | KX356221 | KX356160 | KX356187 | KX345889 |
| *Penelope_superciliaris* | AY367090 | AY659804 | KX356284 | NA | KX345922 | NA | KX356226 | KX356163 | KX356191 | KX345892 |
| *Penelope_jacquacu* | NA | AY659801 | JQ175710 | NA | NA | NA | NA | NA | NA | NA |
| *Penelope_purpurascens* | AY367097 | AY659800 | JQ175718 | KX356269 | KF833521 | KF833695 | KX356225 | NA | KX356190 | NA |
| *Penelope_albipennis* | KX356240 | NA | JN801891 | NA | KX345914 | KX356297 | KX356217 | NA | KX356183 | KX345885 |
| *Penelope_obscura* | AY140742 | AF165474 | JQ175715 | AY140756 | KX345919 | KX356301 | KX356223 | NA | NA | NA |
| *Penelope_pileata* | KX356247 | NA | JQ175717 | KX356268 | KX345920 | KX356300 | KX356224 | KX356162 | KX356189 | KX345891 |
| *Penelope_ochrogaster* | AY367089 | AY367101 | NA | NA | NA | NA | NA | NA | NA | NA |
| *Penelope_dabbenei* | KX356241 | KX356275 | KX356281 | KX356263 | KX345915 | KX356303 | KX356218 | KX356158 | KX356184 | KX345886 |
| *Penelope_jacucaca* | KX356243 | KX356276 | KX356282 | KX356265 | KX345916 | KX356298 | KX356219 | KX356159 | KX356185 | KX345887 |
| *Pipile_pipile* | AY367094 | AY367106 | JQ175861 | NA | KX345925 | KX356299 | KX356229 | KX356166 | KX356195 | KX345895 |
| *Pipile_cumanensis* | AY367099 | AY659798 | NA | KX356271 | KX345924 | NA | NA | NA | KX356194 | KX345894 |
| *Pipile_cujubi* | AY367092 | AY659799 | NA | KX356270 | NA | NA | KX356228 | KX356165 | KX356193 | KX345893 |
| *Pipile_jacutinga* | AY140744 | AF165476 | AF165500 | AY140758 | NA | NA | NA | NA | NA | NA |
| *Aburria_aburri* | AY140740 | AY354489 | JN801479 | AY140754 | KX345896 | NA | KX356196 | KX356143 | KX356167 | NA |
| *Ortalis_cinereiceps* | KX356253 | KX356273 | KF799991 | KX356258 | KX345907 | KX356295 | KX356209 | KX356151 | KX356176 | KX345879 |
| *Ortalis_garrula* | NA | AY659780 | NA | NA | NA | NA | NA | NA | NA | NA |
| *Ortalis_ruficauda* | NA | AY659781 | NA | NA | NA | NA | NA | NA | NA | NA |
| *Ortalis_canicollis* | AY140746 | AF165472 | AF165496 | AY140760 | KX345906 | KX356291 | KX356208 | KX356150 | KX356175 | KX345878 |
| *Ortalis_guttata* | KX356237 | AY659782 | NA | KX356259 | NA | KX356292 | KX356210 | KX356152 | NA | NA |
| *Ortalis_motmot* | NA | AY659778 | KF446138 | KX356260 | KX345909 | KX356307 | KX356212 | KX356154 | KX356178 | KX345881 |
| *Ortalis_erythroptera* | KX356256 | NA | NA | NA | NA | NA | NA | NA | NA | NA |
| *Nothocrax_urumutum* | AY140749 | AY354488 | KM896479 | AY140763 | KX345904 | KX356289 | KX356205 | NA | NA | KX345876 |
| *Crax_rubra* | AY952746 | AY659793 | AY141915 | AY141965 | KX345902 | KX356308 | KX356203 | KX356147 | KX356172 | KC749583 |
| *Crax_alberti* | AY141930 | AY141920 | AY141910 | AY141960 | KX345898 | NA | KX356199 | KX356144 | KX356169 | KX345872 |
| *Crax_daubentoni* | AY141932 | AY141922 | AY141912 | AY141962 | KX345899 | KX356285 | KX356200 | KX356145 | KX356170 | NA |
| *Crax_alector* | AY141931 | EF571188 | JQ174569 | AY141961 | EU302762 | EU738611 | NA | EU737204 | NA | EU302719 |
| *Crax_globulosa* | AY141934 | AY141924 | AY141914 | NA | KX345901 | NA | KX356202 | KX356146 | KX356171 | KX345873 |
| *Crax_fasciolata* | AY141933 | AY659790 | JQ174570 | NA | KX345900 | KX356286 | KX356201 | NA | NA | NA |
| *Crax_blumenbachii* | AY140747 | AY659791 | AF165492 | AY140761 | NA | NA | NA | NA | NA | NA |
| *Mitu_tomentosum* | AY141938 | AY659787 | JQ175400 | AY141968 | NA | NA | NA | NA | NA | NA |
| *Mitu_salvini* | AY141937 | AY659785 | EU525438 | AY141967 | NA | NA | NA | NA | NA | NA |
| *Mitu_tuberosum* | AY140748 | AY354484 | EU525441 | AY140762 | KX345903 | KX356287 | KX356204 | KX356148 | KX356173 | KX345875 |
| *Mitu_mitu* | AY141936 | AY098552 | AY141916 | AY141966 | NA | NA | NA | NA | NA | NA |
| *Pauxi_pauxi* | AY140750 | AY354486 | AF165497 | AY140764 | NA | KX356288 | KX356216 | KX356157 | KX356182 | KX345884 |
| *Pauxi_unicornis* | AY141939 | AY659786 | AY141919 | AY141969 | NA | NA | NA | NA | NA | NA |

**Melipona**

| **Specie** | **Coi** | **16s** | **Rna-pol2** | **Ef1-alpha** | **Argk** |
| --- | --- | --- | --- | --- | --- |
| *Melipona_costaricaensis* | EU163129 | EU162954 | EU162885 | EU163207 | EU163048 |
| *Melipona_solari* | EU163160 | EU162994 | EU162915 | EU163247 | EU163079 |
| *Melipona_ogilviei* | EU163140 | EU162974 | EU162896 | EU163227 | EU163059 |
| *Melipona_micheneri* | EU163139 | EU162973 | EU162895 | EU163226 | EU163058 |
| *Melipona_quadrifasciata* | EU163150 | AF343100 | EU162887 | EU163218 | EU163069 |
| *Melipona_mandacaia* | EU163156 | EU162990 | EU162911 | EU163243 | EU163075 |
| *Melipona_asilvai* | EU163157 | EU162991 | EU162912 | EU163244 | EU163076 |
| *Melipona_melanopleura* | EU163113 | EU162946 | EU162866 | EU163197 | EU163029 |
| *Melipona_panamica* | EU163096 | EU162928 | EU162848 | EU163179 | EU163012 |
| *Melipona_rufiventris* | EU163132 | EU162966 | EU162888 | EU163219 | EU163051 |
| *Melipona_scutellaris* | EU163152 | EU162986 | EU162907 | EU163239 | EU163071 |
| *Melipona_seminigra* | EU163138 | EU162972 | EU162894 | EU163225 | EU163057 |
| *Melipona_fuscopilosa* | EU163136 | EU162970 | EU162892 | EU163223 | EU163055 |
| *Melipona_fulva* | EU163125 | EU162959 | EU162881 | EU163212 | EU163044 |
| *Melipona_lateralis* | EU163144 | EU162978 | EU162900 | EU163231 | EU163063 |
| *Melipona_nebulosa* | EU163146 | EU162980 | NA | EU163233 | EU163065 |
| *Melipona_illota* | EU163167 | EU163001 | EU162921 | EU163254 | FJ042220 |
| *Melipona_crinita* | EU163164 | EU162998 | EU162919 | EU163251 | EU163052 |
| *Melipona_melanoventer* | EU163135 | EU162969 | EU162891 | EU163222 | EU163054 |
| *Melipona_captiosa* | EU163142 | EU162976 | EU162898 | EU163229 | EU163061 |
| *Melipona_fuliginosa* | EU163141 | EU162975 | EU162897 | EU163228 | EU163037 |
| *Melipona_marginata* | EU163153 | EU162987 | EU162908 | EU163240 | EU163072 |
| *Melipona_bicolor* | EU163158 | EU162992 | FJ041919 | EU163245 | FJ042195 |
| *Melipona_amazonica* | EU163166 | EU163000 | NA | EU163253 | EU163085 |
| *Melipona_favosa* | EU163127 | EU162961 | EU162883 | EU163214 | EU163046 |
| *Melipona_compressipes* | EU163137 | AF181589 | EU162893 | EU163224 | EU163056 |
| *Melipona_grandis* | EU163169 | EU163003 | EU162886 | EU163256 | EU163088 |
| *Melipona_triplaridis* | EU163154 | EU162988 | EU162909 | EU163241 | EU163073 |
| *Melipona_quinquefasciata* | EU163155 | EU162989 | EU162910 | EU163242 | EU163074 |
| *Melipona_beecheii* | EU163126 | EU162960 | EU162882 | EU163213 | EU163045 |
| *Melipona_illustris* | EU163145 | EU162979 | EU162901 | EU163232 | EU163064 |
| *Melipona_bradleyi* | NA | FJ041921 | NA | FJ042298 | FJ042197 |
| *Melipona_capixaba* | JN315064 | NA | NA | NA | NA |
| *Melipona_colimana* | JX869619 | JX869598 | NA | NA | NA |
| *Melipona_eburnea* | NA | FJ041935 | NA | FJ042312 | FJ042211 |
| *Melipona_fasciata* | JX869623 | JX869600 | NA | FJ042323 | FJ042222 |
| *Melipona_fasciculata* | NA | FJ041946 | NA | FJ042324 | FJ042223 |
| *Melipona_flavolineata* | KP708582 | NA | NA | NA | NA |
| *Melipona_orbignyi* | FJ975767 | NA | NA | NA | NA |
| *Scaptotrigona* | JQ783156 | L22900 | EU162854 | GU244963 | EU163018 |
| *Trigona* | AF214669 | L22901 | EU162858 | EU049789 | EU184829 |
| *Cephalotrigona* | EU163161 | EU162995 | EU162916 | EU184771 | EU184830 |
| *Geotrigona* | EU163112 | EU162945 | EU162865 | DQ813116 | EU163028 |
| *Plebeia_franki* | EU163098 | EU162930 | EU162850 | EU163181 | EU163014 |
| *Friesella_schrotkyii* | EU163103 | EU162936 | EU162856 | EU163187 | EU163020 |
| *Frieseomelitta* | EU163104 | FJ041924 | EU162857 | EU163188 | EU163021 |
| *Lestrimelitta* | EU163111 | EU162944 | EU162864 | AY208287 | EU163027 |
| *Tetragonisca* | KF224897 | FJ042001 | EU162849 | EU163180 | EU163013 |
| *Nannotrigona* | EU163100 | FJ041953 | EU162853 | EU163184 | EU163017 |
| *Nogueirapis_mirandula* | NA | EU162947 | EU162867 | EU163198 | EU163030 |
| *Meliwillea_bivea* | EU163114 | AF343108 | EU162868 | EU163199 | EU163031 |
| *Meliponula* | EU163118 | EU162951 | EU162873 | EU163204 | EU163036 |
| *Bombus* | JQ769073 | AF364824 | NA | NA | AY739533 |
| *Apis_cerana* | KJ755628 | HQ318940 | NA | EU184774 | EU163040 |
| *Apis_koschevnikovi* | AY754732 | EU162942 | EU162863 | EU163193 | EU163025 |
| *Apis_mellifera* | KR793809 | JF825886 | NA | NA | NA |
| *Apis_dorsata* | KT960840 | KU752359 | NA | NA | AY267178 |
| *Apis_andreniformis* | AB284158 | KU212301 | EU162879 | AY721702 | EU163042 |
| *Apis_florea* | AB284150 | KU752357 | NA | NA | EU184831 |
| *Euglossa_asarophora* | EU421496 | NA | EU421248 | EU163171 | EU421628 |
| *Euglossa_mixta* | EU163094 | NA | EU421309 | EU421436 | NA |
| *Euglossa_villosa* | EU421556 | NA | EU421301 | EU421428 | EU163009 |
| *Euglossa_decorata* | EU421505 | NA | EU162841 | EU163172 | EU421636 |
| *Aglae_caerulea* | EU421542 | EU162926 | EU421289 | EU421413 | EU163007 |
| *Eufriesea_caerulescens* | EU421584 | NA | EU162847 | AY208283 | EU421711 |
| *Eulaema_peruviana* | AJ581111 | AJ581092 | EU421290 | EU421414 | EU163008 |
| *Exaerete_azteca* | EU421557 | EU162927 | EU421302 | EU421429 | EU163010 |
| *Centris* | DQ225328 | EU162948 | EU162869 | EU163200 | EU163032 |
| *Epicharis* | EU163115 | NA | GU245359 | EU163201 | EU163033 |

**Cebidae**

| **Specie** | **Sry** | **Bdnf** | **Rag1** | **Rag2** | **16s** | **Mc1r** | **Cytb** | **Beta2-microglobulin** | **Coii** | **Dmrt1** | **Fbn1** | **Abca1** | **Adora3** | **Aff2** |
| --- | --- | --- | --- | --- | --- | --- | --- | --- | --- | --- | --- | --- | --- | --- |
| *Aotus* | AF338375 | HM763844 | HM759104 | HM758932 | AB107211 | AY205129 | HQ005496 | AF042145 | AF352260 | HM762509 | HM761928 | HM765414 | HM765178 | HM765068 |
| *Lagothrix* | HM757996 | HM763833 | HM759086 | HM758914 | U39005 | NA | KR902423 | AH006721 | HM057599 | HM762589 | HM761911 | HM765336 | HM765159 | HM764984 |
| *Brachyteles* | NA | NA | HM759084 | HM758912 | DQ078115 | NA | AY065906 | AH006720 | AF216253 | HM762519 | NA | HM765272 | HM765157 | HM764912 |
| *Ateles* | DQ976605 | HM763850 | AY065918 | HM758938 | AB116026 | AB296241 | KR902384 | AH006727 | AF216249 | HM762505 | HM761933 | HM765262 | HM765184 | HM764897 |
| *Cacajao* | HM758004 | HM763852 | HM759113 | HM758943 | NA | NA | EU560418 | AH006724 | NA | HM762530 | HM761935 | HM765283 | HM765187 | HM764926 |
| *Chiropotes* | NA | HM763856 | HM759121 | HM758950 | NA | NA | FJ531667 | AH006723 | NA | HM762677 | NA | HM765295 | HM765195 | HM765071 |
| *Pithecia* | NA | FJ648367 | HM759140 | HM758971 | U39007 | NA | KR902426 | AH006729 | JN161059 | HM762634 | HM761960 | HM765380 | HM765215 | HM765029 |
| *Callicebus* | HM758005 | HM763866 | HM759137 | HM758970 | AB107212 | NA | AF289988 | AH006728 | JN161057 | HM762527 | HM761959 | HM765284 | HM765210 | HM764941 |
| *Alouatta* | DQ875683 | HM763841 | AY065919 | HM758927 | U38997 | AY205132 | AY374376 | AH006717 | AF054296 | HM762515 | HM761922 | HM765267 | HM765171 | HM764906 |
| *Cebus_albifrons* | AF338385 | JN633376 | HM759115 | HM758944 | NA | AY205128 | FJ529109 | AH006509 | NA | HM762675 | HM761936 | HM765415 | KU694339 | HM765069 |
| *Cebus_apella* | AF338387 | NA | HM759116 | HM758945 | U39003 | NA | FJ529104 | AH006508 | AF181088 | HM762524 | HM761937 | HM765278 | HM765190 | HM764920 |
| *Cebus_capucinus* | AF338388 | HM763853 | NA | HM758946 | NA | NA | FJ529110 | NA | JF735240 | HM762532 | HM761938 | HM765285 | HM765191 | HM764928 |
| *Cebus_nigritus* | NA | NA | HM759118 | HM758948 | NA | NA | KR528406 | NA | AF181088 | NA | HM761940 | HM765308 | NA | HM764953 |
| *Cebus_olivaceus* | AF338389 | HM763854 | HM759117 | HM758947 | NA | NA | FJ529107 | AH006733 | NA | HM762551 | HM761939 | HM765303 | HM765192 | HM764948 |
| *Cebus_xanthosternos* | NA | NA | HM759119 | NA | NA | NA | JN835288 | NA | NA | NA | NA | NA | NA | NA |
| *Saimiri_boliviensis* | HM758016 | NA | HM759129 | HM758961 | DQ334826 | NA | NA | NA | AF396460 | HM762645 | HM761953 | NA | HM765204 | HM765040 |
| *Saimiri_oerstedii* | HM758017 | HM763864 | HM759130 | HM758962 | NA | AY205127 | KM234547 | NA | NA | HM762657 | HM761954 | HM765400 | HM765205 | HM765052 |
| *Saimiri_sciureus* | HM758018 | HM763865 | HM759131 | HM758963 | AB107214 | AB296239 | HQ005511 | AH007458 | U36848 | HM762658 | HM761955 | HM765401 | HM765206 | HM765053 |
| *Saimiri_ustus* | NA | NA | NA | NA | NA | NA | EU232707 | NA | NA | NA | NA | NA | NA | NA |
| *Saimiri_vanzolinii* | NA | NA | NA | NA | NA | NA | KM234514 | NA | NA | NA | NA | NA | NA | NA |
| *Leontopithecus_chrysomelas* | AF338370 | HM763839 | HM759094 | HM758923 | NA | AY205116 | KR528398 | NA | NA | HM762585 | HM761918 | HM765332 | HM765168 | HM764980 |
| *Leontopithecus_chrysopygus* | AF338371 | NA | NA | NA | NA | AY205115 | NA | AH006736 | NA | NA | NA | NA | NA | NA |
| *Leontopithecus_rosalia* | AF338373 | HM763840 | HM759095 | HM758924 | U39006 | AY205114 | KR528404 | NA | NA | HM762590 | HM761919 | HM765337 | HM765169 | HM764985 |
| *Callimico_goeldii* | AF338383 | AY011480 | HM759087 | HM758915 | U39000 | AY205121 | NA | AH006734 | AY118175 | HM762538 | HM761912 | HM765291 | HM765160 | HM764934 |
| *Callithrix_aurita* | AF338392 | NA | NA | HM758917 | NA | NA | KR528400 | AH008973 | AY118188 | NA | NA | NA | HM765162 | HM764917 |
| *Callithrix_geoffroyi* | AF338378 | HM763835 | HM759089 | HM758918 | NA | AY205119 | KU253509 | NA | AY118192 | HM762537 | HM761914 | HM765290 | HM765163 | HM764933 |
| *Callithrix_kuhlii* | AF338380 | NA | HM759091 | HM758920 | NA | NA | KU253511 | AH006715 | AY118193 | NA | NA | HM765297 | HM765165 | HM764940 |
| *Callithrix_penicillata* | AF338381 | HM763837 | HM759092 | HM758921 | NA | NA | NA | AH007459 | AY118196 | HM762552 | HM761916 | HM765304 | HM765166 | HM764949 |
| *Callithrix_jacchus* | AF338379 | HM763836 | HM759090 | HM758919 | U39001 | AY205120 | AY434079 | AH008974 | AY321457 | HM762543 | HM761915 | HM765296 | NM_001302791 | XM_017968309 |
| *Cebuella_pygmaea* | AF338382 | HM763838 | HM759093 | HM758922 | U29002 | NA | NA | AH006726 | NA | NA | NA | NA | NA | NA |
| *Mico_argentatus* | NA | HM763834 | HM759088 | HM758916 | NA | AY205118 | AF245065 | NA | AY118183 | HM762525 | HM761913 | HM765279 | HM765161 | HM764921 |
| *Mico_emiliae* | NA | NA | NA | NA | FJ769146 | NA | L44587 | AH006735 | AY118178 | NA | NA | NA | NA | NA |
| *Mico_humeralifer* | NA | NA | HM759096 | HM758925 | NA | NA | AF245052 | AH007460 | AY118184 | NA | NA | HM765345 | HM765170 | HM764994 |
| *Mico_mauesi* | NA | NA | NA | NA | FJ769147 | NA | AF245051 | NA | AY118187 | NA | NA | NA | NA | NA |
| *Mico_saterei* | NA | NA | NA | NA | NA | NA | NA | NA | AY118180 | NA | NA | NA | NA | NA |
| *Mico_chrysoleuca* | NA | NA | NA | NA | NA | NA | KR528411 | NA | NA | NA | NA | NA | NA | NA |
| *Saguinus_nigricollis* | NA | NA | NA | NA | NA | NA | HM368075 | NA | NA | NA | NA | NA | NA | NA |
| *Saguinus_fuscicollis* | HM758012 | HM763858 | HM759123 | HM758953 | NA | AY205123 | NA | AH008977 | NA | HM762648 | NA | HM765391 | HM765197 | HM765043 |
| *Saguinus_tripartitus* | NA | NA | NA | NA | NA | NA | HM368006 | NA | NA | NA | NA | NA | NA | NA |
| *Saguinus_imperator* | NA | NA | NA | HM758955 | EU497287 | AY205122 | HM368020 | AH006731 | NA | HM762651 | HM761946 | HM765394 | NA | HM765046 |
| *Saguinus_labiatus* | NA | HM763860 | HM759125 | HM758956 | EU497289 | NA | HM367998 | NA | NA | HM762652 | HM761947 | HM765395 | NA | HM765047 |
| *Saguinus_inustus* | NA | NA | NA | NA | NA | NA | KM370859 | NA | NA | NA | NA | NA | NA | NA |
| *Saguinus_mystax* | HM758014 | HM763862 | HM759127 | HM758959 | EU497294 | NA | HM367983 | AH008978 | NA | HM762655 | HM761951 | HM765398 | HM765202 | HM765050 |
| *Saguinus_leucopus* | NA | NA | NA | NA | EU497286 | NA | NA | NA | NA | NA | NA | NA | NA | NA |
| *Saguinus_oedipus* | HM758015 | HM763863 | HM759128 | HM758960 | EU497296 | AY205125 | HM368007 | AH006510 | NA | HM762656 | HM761952 | HM765399 | HM765203 | HM765051 |
| *Saguinus_niger* | NA | NA | NA | NA | EU497246 | NA | NA | AH006193 | NA | NA | NA | NA | NA | NA |
| *Saguinus_midas* | AF338391 | HM763861 | HM759126 | HM758958 | EU497273 | AY205126 | AJ489760 | AH006716 | NA | HM762653 | HM761950 | HM765396 | HM765200 | HM765048 |
| *Saguinus_bicolor* | HM758011 | HM763857 | HM759122 | HM758952 | EU497280 | NA | NA | AH008975 | NA | HM762644 | HM761944 | HM765388 | HM765196 | HM765039 |
| *Saguinus_martinsi* | NA | NA | NA | NA | EU497276 | NA | NA | AH008976 | NA | HM762659 | HM761948 | HM765402 | NA | HM765054 |
| *Saguinus_geoffroyi* | NA | HM763859 | NA | NA | U39008 | AY205124 | NA | NA | NA | NA | NA | NA | NA | NA |
| *Saguinus_graellsi* | NA | NA | NA | NA | NA | NA | HM368035 | NA | NA | NA | NA | NA | NA | NA |
| *Saguinus_melanoleucus* | NA | NA | NA | NA | NA | NA | HM368078 | NA | NA | NA | NA | NA | NA | NA |
| *Callibella_humilis* | NA | NA | NA | NA | FJ769145 | NA | NA | NA | NA | NA | NA | NA | NA | NA |

**Echimyidae**

| **Specie** | **Cytb** | **Rag1** | **16s** | **12s** | **Ghr** | **Coi** | **Vwf** |
| --- | --- | --- | --- | --- | --- | --- | --- |
| *Capromys_pilorides* | AF422915 | KM013988 | KM013971 | KM013959 | AF433950 | NA | AJ251142 |
| *Myocastor_coypus* | EU544663 | JN414955 | AY011155 | AY012123 | NA | NA | AJ251140 |
| *Chinchilla_lanigera* | AF244382 | KF590658 | AY062170 | AF520696 | AY701337 | GU130595 | NA |
| *Ctenomys_boliviensis* | AF007040 | NA | NA | U12446 | JN414757 | JQ341048 | NA |
| *Octodontomys_gliroides* | AF370706 | KF590663 | NA | AF520684 | AF520664 | GQ121084 | KF590672 |
| *Octodon_bridgesi* | KJ742651 | KJ742676 | NA | AF520677 | AF520646 | NA | KJ742611 |
| *Abrocoma_cinerea* | AF244388 | NA | NA | AF520666 | AF520643 | NA | NA |
| *Abrocoma_bennettii* | AF244387 | NA | NA | NA | FJ855213 | NA | JN415073 |
| *Dactylomys_boliviensis* | L23339 | EU313299 | AF422909 | AF422875 | KF590679 | NA | AJ849307 |
| *Dactylomys_dactylinus* | L23335 | EU313301 | AF422908 | AF422874 | KF590681 | NA | KF590667 |
| *Dactylomys_peruanus* | EU313207 | NA | NA | NA | NA | NA | NA |
| *Kannabateomys_amblyonyx* | AF422917 | NA | AF422884 | AF422850 | NA | NA | AJ849310 |
| *Olallamys_albicauda* | KF590697 | NA | NA | NA | KF590691 | NA | KF590673 |
| *Callistomys_pictus* | KJ742659 | KJ742677 | NA | KJ742594 | KJ742627 | NA | KJ742614 |
| *Diplomys_labilis* | KJ742660 | KJ742685 | NA | NA | KJ742636 | NA | KJ742613 |
| *Diplomys_rufodorsalis* | KJ742664 | NA | NA | NA | NA | NA | NA |
| *Echimys_chrysurus* | L23341 | EU313303 | AF422911 | AY093663 | FJ855215 | JF458603 | AJ251141 |
| *Echimys_semivillosus* | KJ742662 | KJ742687 | NA | NA | NA | JF458604 | KJ742616 |
| *Isothrix_bistriata* | L23355 | EU313311 | AF422907 | AF422873 | FJ855216 | NA | AJ849308 |
| *Isothrix_negrensis* | EU313221 | NA | NA | NA | NA | NA | NA |
| *Isothrix_pagurus* | EU313226 | KF590661 | NA | KF590703 | KF590684 | NA | KF590670 |
| *Isothrix_sinnamariensis* | AY745734 | EU313313 | NA | KF590704 | KF590686 | JF458606 | AJ849309 |
| *isothrix_barbarabrownae* | EU313214 | EU313304 | NA | KF590701 | KF590682 | NA | KF590668 |
| *Isothrix_orinoci* | EU313225 | KF590660 | NA | KF590702 | KF590683 | NA | KF590669 |
| *Makalata_didelphoides* | L23363 | EU313320 | AF422912 | KJ742600 | KJ742639 | JF458629 | JF297707 |
| *Makalata_grandis* | KF590699 | EU313336 | NA | NA | KF590694 | NA | KF590676 |
| *Makalata_macrura* | EU302703 | EU313330 | AF422913 | AF422879 | KF590687 | NA | AJ849312 |
| *Makalata_occasius* | KJ742661 | NA | NA | NA | KJ742637 | NA | NA |
| *Makalata_rhipidura* | KJ742663 | KJ742686 | NA | NA | KJ742638 | NA | KJ742617 |
| *Phyllomys_blainvillii* | JF297836 | KF590664 | NA | KF590706 | KF590692 | JF297686 | JF297735 |
| *Phyllomys_brasiliensis* | EF608182 | NA | NA | AY093666 | NA | JF297680 | JF297729 |
| *Phyllomys_dasythrix* | EF608185 | KJ742689 | NA | KJ742605 | KJ742641 | JF297660 | JF297709 |
| *Phyllomys_lamarum* | EF608181 | NA | NA | NA | NA | JF297682 | JF297731 |
| *Phyllomys_lundi* | EF608183 | NA | NA | NA | NA | JF297672 | JF297721 |
| *Phyllomys_mantiqueirensis* | EF608179 | NA | NA | NA | NA | JF297671 | JF297720 |
| *Phyllomys_nigrispinus* | EF608184 | NA | NA | NA | NA | JF297666 | JF297719 |
| *Phyllomys_pattoni* | EF608187 | KJ742690 | NA | KJ742606 | KJ742642 | JF297704 | JF297754 |
| *Phyllomys_unicolor* | EF608188 | NA | NA | NA | NA | NA | NA |
| *Carterodon_sulcidens* | KJ742666 | KJ742678 | NA | KJ742596 | KJ742640 | NA | KJ742615 |
| *Clyomys_laticeps* | AF422918 | KJ742679 | AF422885 | KJ742597 | KJ742628 | NA | AJ849306 |
| *Euryzygomatomys_spinosus* | EU544667 | KJ742680 | NA | NA | KJ742629 | GU938885 | AJ849319 |
| *Hoplomys_gymnurus* | AF422922 | JN414965 | AF422896 | AF520668 | JN414758 | NA | JN415080 |
| *Lonchothrix_emiliae* | EU313229 | NA | AF422891 | AF422857 | NA | NA | NA |
| *Mesomys_hispidus* | KF590696 | EU313322 | AF422895 | KF590705 | KF590688 | HQ919652 | KF590671 |
| *Mesomys_occultus* | NA | EU313331 | AF422893 | AF422859 | KF590689 | NA | NA |
| *Mesomys_stimulax* | KJ742667 | KJ742674 | NA | KJ742603 | KJ742630 | NA | KJ742618 |
| *Proechimys_cuvieri* | AY206633 | KF590665 | NA | KF590707 | KF590693 | JF458720 | KF590675 |
| *Proechimys_guyannensis* | NA | NA | NA | KX381547 | NA | EU096897 | NA |
| *Proechimys_hoplomyoides* | NA | NA | NA | NA | NA | EU095484 | NA |
| *Proechimys_longicaudatus* | NA | KJ742681 | NA | U12447 | KJ742643 | NA | KJ742619 |
| *Proechimys_quadruplicatus* | AF308435 | NA | NA | NA | NA | EU095487 | AJ849313 |
| *Proechimys_roberti* | EU544666 | NA | NA | NA | NA | NA | NA |
| *Proechimys_simonsi* | U35414 | EU313333 | AF422898 | AF422864 | KJ742631 | EU095486 | AJ849320 |
| *Proechimys_steerei* | NA | NA | NA | NA | NA | JF459060 | NA |
| *Thrichomys_apereoides* | EU544668 | EU313335 | AF422890 | AF422856 | JX515325 | NA | AJ849315 |
| *Thrichomys_inermis* | JX459887 | NA | NA | NA | NA | NA | NA |
| *Thrichomys_pachyurus* | AY083340 | NA | NA | NA | NA | NA | NA |
| *Trinomys_albispinus* | KM014008 | KM013992 | KM013976 | KM013964 | KM013987 | NA | KM014003 |
| *Trinomys_dimidiatus* | AF194296 | KJ742682 | AF422901 | AF422867 | NA | NA | KJ742620 |
| *Trinomys_eliasi* | KJ707247 | NA | AF422903 | AF422869 | NA | NA | NA |
| *Trinomys_gratiosus* | KJ707248 | NA | NA | NA | NA | NA | NA |
| *Trinomys_iheringi* | EU544664 | EU313338 | AF422902 | AF422868 | KF590695 | NA | KF590677 |
| *Trinomys_moojeni* | KF562097 | NA | NA | NA | NA | NA | NA |
| *Trinomys_paratus* | U35165 | NA | AF422900 | AF422866 | JX515330 | NA | AJ849316 |
| *Trinomys_setosus* | AF422924 | NA | AF422906 | AF422872 | JX515329 | NA | AJ849317 |
| *Trinomys_yonenagae* | AF194295 | NA | AF422899 | AF422865 | JX515328 | NA | AJ849318 |

**Stenodermatinae**

| **Specie** | **Cytb** | **Rag2** | **Nd2** | **Coi** | **Plcb4** | **Atp7** | **Bdnf** | **Thy** |
| --- | --- | --- | --- | --- | --- | --- | --- | --- |
| *Rhinophylla_alethina* | AF187027 | NA | NA | JF449072 | NA | NA | NA | NA |
| *Rhinophylla_fischerae* | AF187032 | KF569351 | NA | JF449110 | KF569329 | NA | NA | KF569455 |
| *Carollia_subrufa* | AF187024 | NA | NA | JF448015 | KM362009 | NA | NA | NA |
| *Carollia_castanea* | AF512006 | FN641676 | NA | JF448773 | KF569314 | NA | KF569466 | KF569450 |
| *Glyphonycteris_sylvestris* | AY380746 | AF316471 | NA | KX910802 | KM362017 | NA | NA | NA |
| *Trinycteris_nicefori* | AY380749 | AF316469 | NA | JF456028 | KC783108 | NA | KC783000 | KC783252 |
| *Lonchophylla_hesperia* | KF815310 | KM362062 | NA | NA | KM362022 | NA | KM361983 | NA |
| *Lonchophylla_dekeyseri* | NA | NA | NA | NA | KM362021 | KM361948 | KM361982 | NA |
| *Lionycteris_spurrelli* | KF815304 | AF316455 | NA | JF454738 | KC783075 | NA | KC782968 | KC783226 |
| *Platalina_genovensium* | KF815311 | NA | NA | NA | NA | NA | NA | NA |
| *Lonchophylla_chocoana* | AF423092 | NA | NA | JF448854 | KF569321 | NA | KF569473 | KF569453 |
| *Lonchophylla_mordax* | AF423095 | KC783116 | NA | JF448855 | KM362020 | NA | NA | NA |
| *Centurio_senex* | AY604444 | AF316438 | NA | JF447241 | KF569315 | NA | NA | KM362119 |
| *Sturnira_lilium* | DQ312398 | KC754302 | KC753968 | JF459281 | KC783105 | KC783048 | KC782996 | KC783248 |
| *Sturnira_tildae* | KC753897 | DQ903847 | NA | JF455952 | KF569331 | KF569434 | KF569480 | KF569452 |
| *Sturnira_ludovici* | KC753807 | KC754272 | KC753925 | JN659773 | KF569334 | KF569432 | NA | NA |
| *Sturnira_luisi* | KC753815 | KC754280 | KC753933 | JN659882 | KM362042 | NA | KM361996 | KM362139 |
| *Sturnira_erythromos* | FJ154179 | FJ154377 | FJ154245 | JN659617 | NA | NA | NA | NA |
| *Sturnira_parvidens* | KC753873 | KC754337 | KC753994 | KX756039 | NA | NA | NA | NA |
| *Sturnira_magna* | KC753820 | KC754283 | KC753938 | JN659896 | KF569332 | KF569433 | NA | NA |
| *Sturnira_bidens* | AF435200 | NA | KC753900 | JN659567 | NA | NA | NA | NA |
| *Sturnira_oporaphilum* | KC753855 | KC754318 | KC753975 | NA | KM362043 | KM361963 | KM361997 | KM362140 |
| *Sturnira_hondurensis* | KC753827 | KC754288 | KC753946 | NA | NA | NA | NA | NA |
| *Sturnira_bogotensis* | KC753787 | KC754252 | KC753905 | NA | KF569333 | KF569431 | NA | KF569451 |
| *Sturnira_mordax* | KC753824 | KC754287 | KC753943 | NA | NA | NA | NA | NA |
| *Sturnira_burtonlimi* | NA | NA | NA | KX814421 | NA | NA | NA | NA |
| *Sturnira_adrianae* | KY366229 | NA | NA | NA | NA | NA | NA | NA |
| *Sturnira_perla* | NA | NA | NA | JN659911 | NA | NA | NA | NA |
| *Sturnira_aratathomasi* | AF435252 | NA | KC753899 | NA | NA | NA | NA | NA |
| *Sturnira_nana* | AF435252 | NA | KC753899 | NA | NA | NA | NA | NA |
| *Uroderma_bilobatum* | L28941 | AF316491 | NA | JF456031 | NA | KC783052 | NA | NA |
| *Uroderma_magnirostris* | AY169957 | FJ154378 | FJ154246 | JF456032 | KF569335 | KF569435 | KF569481 | KF569449 |
| *Vampyriscus_bidens* | FJ154181 | FJ154379 | FJ154247 | JF456128 | KM362046 | NA | KM361999 | KM362142 |
| *Vampyriscus_nymphaea* | DQ312418 | KF569357 | NA | JF448146 | KF569336 | KF569436 | KF569482 | NA |
| *Vampyriscus_brocki* | DQ312421 | KM362070 | NA | JF456129 | KM362047 | KM361967 | NA | NA |
| *Vampyressa_thyone* | DQ312431 | KF569358 | NA | JN312368 | KF569337 | KF569437 | KF569483 | KF569448 |
| *Vampyressa_melissa* | FJ154185 | FJ154383 | FJ154251 | NA | NA | NA | NA | EU371980 |
| *Vampyressa_pusilla* | DQ312428 | DQ903844 | NA | JF448148 | KM362048 | KM361968 | KM362000 | EU371990 |
| *Chiroderma_villosum* | L28943 | FJ154319 | FJ154187 | JF454586 | NA | NA | NA | EU371975 |
| *Chiroderma_trinitatum* | L28942 | KF569345 | NA | JF454562 | KF569316 | NA | NA | NA |
| *Chiroderma_doriae* | AY169958 | KM362056 | NA | JF448016 | KM362010 | KM361941 | KM361977 | NA |
| *Chiroderma_salvini* | L28939 | KM362058 | NA | JF446777 | KM362012 | KM361943 | KM361979 | KM362121 |
| *Chiroderma_improvisum* | L28938 | KM362057 | NA | NA | KM362011 | KM361942 | KM361978 | KM362120 |
| *Mesophylla_macconnelli* | FJ154122 | FJ154320 | AY504555 | JF454947 | KF569324 | NA | NA | EU371977 |
| *Vampyrodes_caraccioli* | FJ154184 | FJ154382 | FJ154250 | JF456147 | KC783110 | KC783053 | KC783002 | EU371991 |
| *Vampyrodes_major* | HQ637422 | NA | NA | NA | NA | NA | NA | NA |
| *Platyrrhinus_helleri* | FJ154141 | FJ154339 | FJ154206 | JF455411 | KC783100 | KC783043 | KC782991 | KC783244 |
| *Platyrrhinus_aurarius* | FJ154129 | FJ154327 | FJ154195 | JF455410 | KM362031 | KM361955 | KM361988 | KM362131 |
| *Platyrrhinus_lineatus* | FJ154173 | FJ154358 | FJ154226 | JF446382 | KM362035 | KM361959 | KM361991 | KF569447 |
| *Platyrrhinus_infuscus* | FJ154151 | FJ154349 | FJ154217 | JF449066 | KF569326 | NA | NA | NA |
| *Platyrrhinus_dorsalis* | FJ154139 | FJ154337 | FJ154205 | NA | KM362032 | KM361956 | KM361989 | KM362132 |
| *Platyrrhinus_recifinus* | FJ154176 | FJ154374 | FJ154242 | JF446385 | KM362036 | KM361960 | KM361992 | KM362136 |
| *Platyrrhinus_brachycephalus* | FJ154132 | FJ154330 | FJ154198 | JF447853 | KF569327 | KF569428 | KF569477 | KF569446 |
| *Platyrrhinus_guianensis* | KJ576932 | KJ576959 | KJ576941 | NA | NA | NA | NA | NA |
| *Platyrrhinus_ismaeli* | FJ154155 | FJ154353 | FJ154221 | NA | KM362034 | KM361958 | KM361990 | KM362134 |
| *Platyrrhinus_matapalensis* | FJ154168 | FJ154366 | FJ154234 | NA | NA | NA | NA | KM362135 |
| *Platyrrhinus_masu* | FJ154164 | FJ154362 | FJ154230 | NA | NA | NA | NA | NA |
| *Platyrrhinus_albericoi* | FJ154124 | FJ154322 | FJ154190 | NA | KM362030 | KM361954 | KM361987 | KM362130 |
| *Platyrrhinus_vittatus* | FJ154178 | FJ154376 | FJ154243 | JF446602 | KM362037 | NA | NA | NA |
| *Platyrrhinus_incarum* | FJ154147 | FJ154344 | FJ154213 | NA | KM362033 | KM361957 | KM381957 | KM362133 |
| *Platyrrhinus_nigellus* | FJ154173 | FJ154371 | FJ154239 | NA | KM362035 | KM361959 | KM361991 | NA |
| *Enchisthenes_hartii* | EU160972 | AF316449 | NA | JF447409 | KC783070 | KC783016 | KC782964 | KC783222 |
| *Ectophylla_alba* | DQ312404 | AF316448 | NA | JF446595 | NA | KF569420 | KF569469 | KF569445 |
| *Sphaeronycteris_toxophyllum* | AY604452 | AF316486 | NA | NA | KF569330 | KF569430 | KF569479 | NA |
| *Pygoderma_bilabiatum* | AY604438 | DQ903839 | NA | NA | KC783103 | KC783046 | KC782994 | KC783247 |
| *Ametrida_centurio* | AY604446 | AF316430 | NA | JF452119 | KF569308 | KF569409 | NA | NA |
| *Ardops_nichollsi* | KJ024748 | AF316434 | NA | NA | NA | NA | NA | KF569438 |
| *Ariteus_flavescens* | KJ024703 | AF316435 | NA | NA | KM362003 | KF569410 | NA | KM362113 |
| *Stenoderma_rufum* | DQ312400 | AF316487 | NA | NA | NA | NA | NA | EU371963 |
| *Phyllops_falcatus* | DQ211651 | AY604453 | NA | NA | NA | NA | NA | NA |
| *Artibeus_lituratus* | EU160833 | DQ985529 | NA | EU161033 | KC783061 | NA | KC782955 | EU371964 |
| *Artibeus_jamaicensis* | DQ869504 | FN641674 | NA | JF459376 | KC783060 | AY011419 | KC782954 | AJ865664 |
| *Artibeus_planirostris* | AY684720 | NA | NA | EU161056 | NA | NA | NA | NA |
| *Artibeus_obscurus* | EU160865 | NA | NA | EU161044 | KC783062 | NA | KC782956 | KC783217 |
| *Artibeus_glaucus* | EU160970 | KF569339 | NA | EU160995 | KF569310 | KF569414 | KF569463 | KF569444 |
| *Artibeus_cinereus* | EU160687 | AF316443 | NA | EU160983 | KM362004 | KF569412 | KF569461 | KF569440 |
| *Artibeus_schwartzi* | DQ869521 | NA | NA | NA | NA | NA | NA | NA |
| *Artibeus_intermedius* | FJ179231 | KM362055 | NA | JF447942 | KM362008 | KM361940 | KM361976 | KM362118 |
| *Artibeus_phaeotis* | FJ179248 | KF569340 | NA | JF498954 | KF569313 | KF569415 | KF569464 | KF569442 |
| *Artibeus_concolor* | EU160951 | AF316432 | NA | EU160986 | KF569309 | KF569413 | KF569462 | KF569443 |
| *Artibeus_bogotensis* | EU805596 | NA | NA | JN312365 | NA | NA | NA | NA |
| *Artibeus_fimbriatus* | EU160723 | DQ985533 | NA | EU160992 | KM362005 | KM361937 | KM361973 | KM362114 |
| *Artibeus_toltecus* | FJ179258 | NA | NA | KX814389 | KM362013 | NA | NA | KM362143 |
| *Artibeus_amplus* | EU160947 | NA | NA | EU160974 | NA | NA | NA | NA |
| *Artibeus_anderseni* | EU160967 | NA | NA | EU160976 | KF569311 | KF569411 | KF569460 | KF569439 |
| *Artibeus_fraterculus* | EU160955 | KM362052 | NA | EU160994 | KM362006 | KM361938 | KM361974 | KM362115 |
| *Artibeus_aztecus* | FJ179238 | NA | NA | JF447913 | NA | NA | NA | NA |
| *Artibeus_hirsutus* | FJ179226 | AF316433 | NA | NA | NA | KC783007 | NA | NA |
| *Artibeus_inopinatus* | FJ179229 | NA | NA | NA | NA | NA | NA | KM362117 |
| *Artibeus_incomitata* | NA | KM362054 | NA | NA | KM362007 | KM361939 | KM361975 | KM362116 |
| *Rhinophylla_pumilio* | AF187031 | AF316484 | NA | JF455665 | KC783104 | KC783047 | KC782995 | EU371960 |

**Table S1.**

| **Group** | **GBIF’s DOI** |
| --- | --- |
| ***Melipona*** | https://doi.org/10.15468/dl.xmxbkx |
| **Echimyidae** | https://doi.org/10.15468/dl.t6v9hn |
| **Cebidae** | https://doi.org/10.15468/dl.qdgb4q |
| ***Rhinella*** | https://doi.org/10.15468/dl.rq7h5r |
| **Stenodermatinae** | https://doi.org/10.15468/dl.qwkkmk |
| **Cracidae** | https://doi.org/10.15468/dl.pvy4y9 |

**Table S2.**

| **Taxon** | **Fossil** | **Age** |
| --- | --- | --- |
| ***Melipona*** | ***Melittosphex burmensis***  (Poinar & Danforth, 2006) | ~ 99.6–93.5 Ma  Late Cretaceous |
|  | ***Proplebeia dominicana***  (Wille & Chandler, 1964) | ~ 20.4–13.8 Ma  Early-Middle Miocene |
|  | ***Apis lithohermaea***  (Engel, 2006) | ~ 15.9–13.8 Ma  Middle Miocene |
|  | ***Euglossa moronei***  (Engel, 1999) | ~ 20.4–13.8 Ma  Early-Middle Miocene |
| **Echimyidae** | ***Draconomys verai****  (Vucetich et al., 2010) | ~ 33.9–28.1 Ma  Early Oligocene |
|  | ***Sallamys woodi****  (Peréz et al., 2018) | ~ 29–21 Ma  Late Oligocene |
|  | ***Paradelphomys fissus****  (Patterson & Pascual, 1968) | ~ 21–17.5 Ma  Early Miocene |
|  | ***Pampamys emmonsae***  (Verzi et al., 1995) | ~ 9–6.8 Ma  Late Miocene |
| **Cebidae** | ***Branisella boliviana*****  (Hoffstetter, 1969; Rosenberger, 1981; Takai et al., 2000) | ~ 29–21 Ma  Late Oligocene |
|  | ***Cebupithecia sarmientoi*****  (Stirton & Savage, 1950) | ~13.8–11.8 Ma  Middle Miocene |
|  | ***Neosaimiri fieldsi***  (Stirton, 1951) | ~13.8–11.8 Ma  Middle miocene |
|  | ***Stirtonia tatacoensis***  (Hershkovitz, 1970) | ~13.8–11.8 Ma  Middle Miocene |
|  | ***Lagonimico conclucatus***  (Kay, 1994) | ~13.8–12.6 Ma  Middle miocene |
|  | ***Cebuella sp.***  (Marivaux et al., 2016; Antoine et al., 2016) | ~11.8–10.6 Ma  Mayoan, Miocene |
| ***Rhinella*** | **Oldest fossil of Bufonidae**  (Baéz & Gasparini, 1979) | ~57 Ma  Late Palaeocene |
|  | ***Rhinella arenarum***  (Tambussi 1998, Peréz et al., 2014) | ~7.2–5.3 Ma  Late Miocene |
| **Stenodermatinae** | ***Cubanycteris silvai***  (Mancina & García-Rivera, 2005) | ~0.126–0.0117 Ma  Quaternary |
|  | ***Phyllops silvai***  (Suárez & Díaz-Franco, 2003) | ~0.126–0.0117 Ma  Quaternary |
|  | ***Palynephyllum antimaster***  (Czaplewski et al., 2003) | ~13.8–11.8 Ma  Middle Miocene |
| **Cracidae** | ***Gallinuloides wyomingensis***  (Eastman, 1900; Weidig, 2010) | ~55.8–50.3 Ma  Early Eocene |
|  | ***Palaeortyx gallica***  (Storch et al., 1996; Mayr et al., 2006) | ~28.4–23.3 Ma  Late Oligocene |
|  | ***Schaubortyx keltica***  (Eastman, 1905; Brodkorb,1964; Mayr et al., 2006) | ~29.3–27.5 Ma  Oligocene |
|  | ***Rhegminornis calobates***  (White, 1942; Wetmore, 1943) | ~20.4–15.9 Ma  Early Miocene |
|  | ***Lophortyx shotwelli***  (Shotwell, 1956; Brodkorb, 1964) | ~10.3–4.9 Ma  Late Miocene |

***** Although *Draconomys verai*, *Sallamys woodi* and *Paradelphomys fissus* are not echimyids rats, but octodontoids they wer used as calibrated points within the outgroup.

** Although *Branisella boliviana* and *Cebupithecia sarmientoi* are not cebid monkeys, they were used as calibrated points within the outgroup.

**Table S3.**

| **Taxon** | **Gene** | **Strict** | **Relaxed** | **Delta** |
| --- | --- | --- | --- | --- |
| **Stenodermatinae** | THY | -2.99E+003 | -2.98E+003 | -11.933 |
|  | RAG2 | -4.38E+003 | -4.37E+003 | -12.917 |
|  | PLCB4 | -1.78E+003 | -1.70E+003 | -83.752 |
|  | ND2 | -1.11E+004 | -1.09E+004 | -215.26 |
|  | CYTB | -2.05E+004 | -2.04E+004 | -82.47 |
|  | COI | -1.29E+004 | -1.36E+004 | 676.21 |
|  | BDNF | -1.49E+003 | -1.49E+003 | -5.819 |
|  | ATP7 | -2.26E+003 | -2.27E+003 | 4.743 |
| **Echimyidae** | 12S | -9.91E+003 | -9.82E+003 | -89.5 |
|  | CYTB | -2.15E+004 | -2.13E+004 | -238.26 |
|  | GHR | -7.42E+003 | -7.19E+003 | -228.412 |
|  | RAG1 | -8.27E+003 | -7.02E+003 | -1253.132 |
|  | VWF | -8.13E+003 | -7.94E+003 | -191.062 |
| **Cebidae** | 16S | -5.82E+003 | -5.80E+003 | -15.684 |
|  | ABCA1 | -5.17E+003 | -5.05E+003 | -119.114 |
|  | ADORA3 | -3.67E+003 | -3.66E+003 | -10 |
|  | AFF2 | -1.90E+004 | -1.90E+004 | -9.31 |
|  | BDNF | -1.27E+003 | -1.25E+003 | -20 |
|  | BETA2 | -6.46E+003 | -6.45E+003 | -10 |
|  | CYTB | -1.16E+004 | -1.16E+004 | -8.44 |
|  | D-LOOP | -1.14E+004 | -1.14E+004 | -31.63 |
|  | DMRT1 | -1.31E+003 | -1.31E+003 | -6.887 |
|  | FBN1 | -1.41E+003 | -1.40E+003 | -10 |
|  | RAG1 | -3.15E+003 | -3.15E+003 | -6 |
|  | RAG2 | -1.55E+003 | -1.54E+003 | -6.278 |
|  | SRY | -3.25E+003 | -3.23E+003 | -13.668 |
| ***Rhinella*** | 12S-TRNA-VAL-16S | -2.69E+004 | -2.68E+004 | -100 |
|  | 16S | -4.87E+003 | -4.84E+003 | -30 |
|  | CYTB | -10543.52 | -10502.04 | -41 |
|  | POMC | -2.90E+003 | -2.89E+003 | -12.117 |
|  | RAG1 | -1.13E+004 | -1.09E+004 | -400 |
|  | RHOD | -1.10E+003 | -1.09E+003 | -10 |
| **Cracidae** | CLTC1 | -4.77E+003 | -4.38E+003 | -386.003 |
|  | CLTC | -6.93E+003 | -6.17E+003 | -757.934 |
|  | COI | -9.58E+003 | -8.68E+003 | -905.836 |
|  | CYTB | -1.05E+004 | -9.73E+003 | -805.309 |
|  | EFF2 | -8.41E+003 | -7.82E+003 | -588.553 |
|  | ND2 | -1.56E+004 | -1.36E+004 | -2008.82 |
|  | ND5 | -7.91E+003 | -7.43E+003 | -473.938 |
|  | RHOD | -9.91E+003 | -8.39E+003 | -1514.413 |
|  | SERPINB14 | -1.03E+004 | -9.09E+003 | -1204.605 |
|  | TFB5 | -1.87E+003 | -1.83E+003 | -40.824 |
| ***Melipona*** | 16S | -9.09E+003 | -8.24E+003 | -852.724 |
|  | ARGK | -1.08E+004 | -9.46E+003 | -1340 |
|  | COI | -1.48E+004 | -1.35E+004 | -1300 |
|  | EF1-ALPHA | -1.46E+004 | -1.20E+004 | -2600 |
|  | RNA-POL2 | -7.73E+003 | -6.13E+003 | -1600 |

**Table S4.**

| **Group** | **Hypothesis** | **Time-periods (Myr)** | **Dispersal rates** | **DEC** |
| --- | --- | --- | --- | --- |
|  |  |  |  | **Likelihood** |
| Stenodermatinae | H0 | NA | 1 | -457.84 |
|  | H1 | Root–10–7 → | 0.25 – 0.50 – 1 | -441.55 |
|  | H2 | Root–11.8–2.5 → | 0.25 – 0.50 – 1 | -439 |
|  | H3 | Root–17–10 → | 0.25 – 0.50 – 1 | -448.61 |
|  | H4 | Root–10–7 → | 0.25 – 0.50 – 0.25 | -465.11 |
| Cracidae | H0 | NA | 1 | -228.86 |
|  | H1 | Root–10–7 → | 0.25 – 0.50 – 1 | -225.14 |
|  | H2 | Root–11.8–2.5 → | 0.25 – 0.50 – 1 | -221.97 |
|  | H3 | Root–17–10 → | 0.25 – 0.50 – 1 | -227.12 |
|  | H4 | Root–10–7 → | 0.25 – 0.50 – 0.25 | -233.38 |
| Echimyidae | H0 | NA | 1 | -231.82 |
|  | H1 | Root–10–7 → | 0.25 – 0.50 – 1 | -233.56 |
|  | H2 | Root–11.8–2.5 → | 0.25 – 0.50 – 1 | NA |
|  | H3 | Root–17–10 → | 0.25 – 0.50 – 1 | -231.54 |
|  | H4 | Root–10–7 → | 0.25 – 0.50 – 0.25 | NA |
| Cebidae | H0 | NA | 1 | -151.63 |
|  | H1 | Root–10–7 → | 0.25 – 0.50 – 1 | -154.46 |
|  | H2 | Root–11.8–2.5 → | 0.25 – 0.50 – 1 | -154.23 |
|  | H3 | Root–17–10 → | 0.25 – 0.50 – 1 | -153.04 |
|  | H4 | Root–10–7 → | 0.25 – 0.50 – 0.25 | -152.85 |
| *Rhinella* | H0 | NA | 1 | -133.37 |
|  | H1 | Root–10–7 → | 0.25 – 0.50 – 1 | -130.83 |
|  | H2 | Root–11.8–2.5 → | 0.25 – 0.50 – 1 | -130.73 |
|  | H3 | Root–17–10 → | 0.25 – 0.50 – 1 | -130.36 |
|  | H4 | Root–10–7 → | 0.25 – 0.50 – 0.25 | -133.39 |
| *Melipona* | M0 | NA | 1 | -210.41 |
|  | H0 | Root–10–7 → | 0.25 – 0.50 – 1 | -212.55 |
|  | H1 | Root–11.8–2.5 → | 0.25 – 0.50 – 1 | -211.18 |
|  | H2 | Root–17–10 → | 0.25 – 0.50 – 1 | -215.67 |
|  | H3 | Root–10–7 → | 0.25 – 0.50 – 0.25 | -211.89 |

**Figure S1.**

**
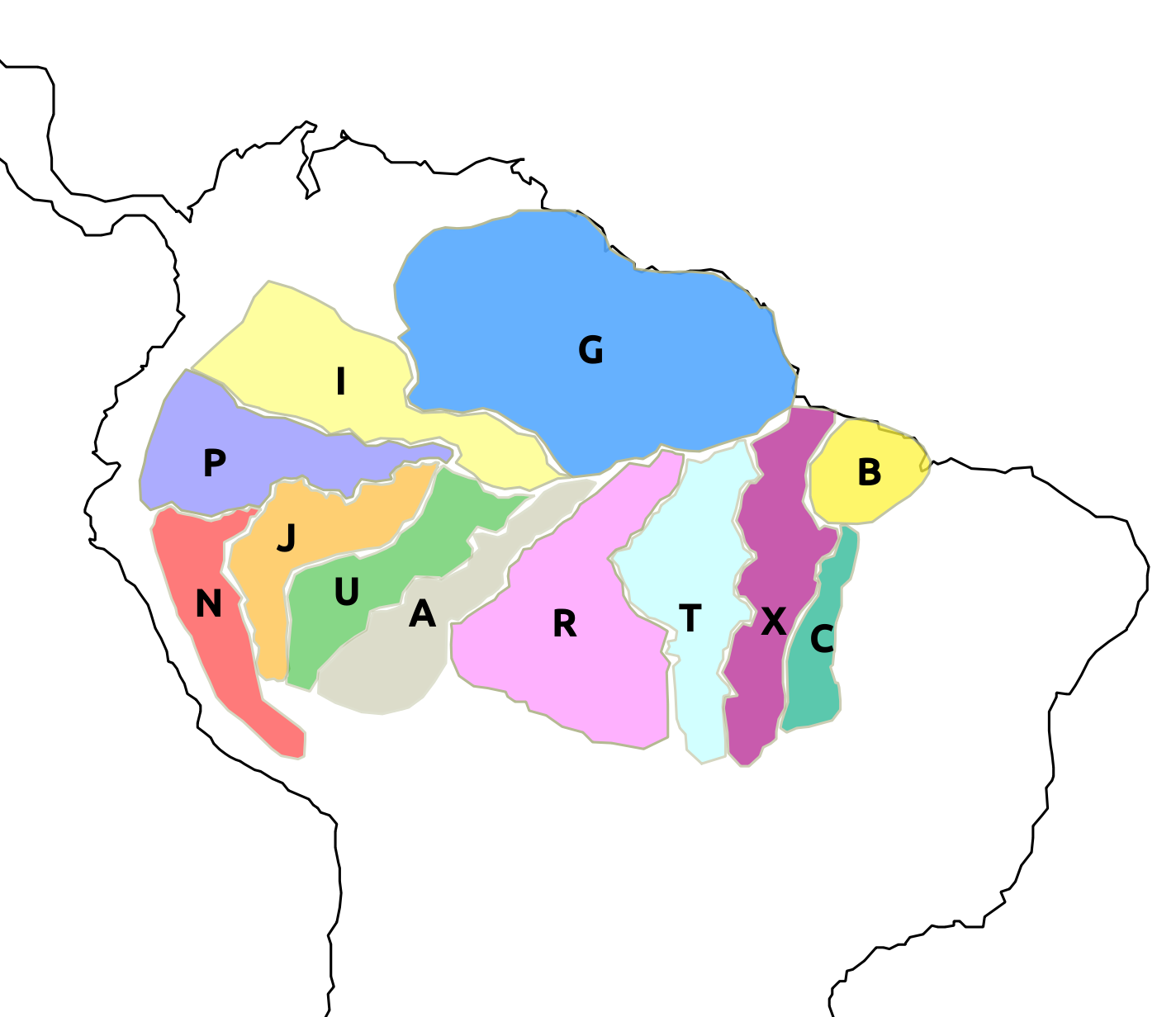
**

**Figure S2.**

**
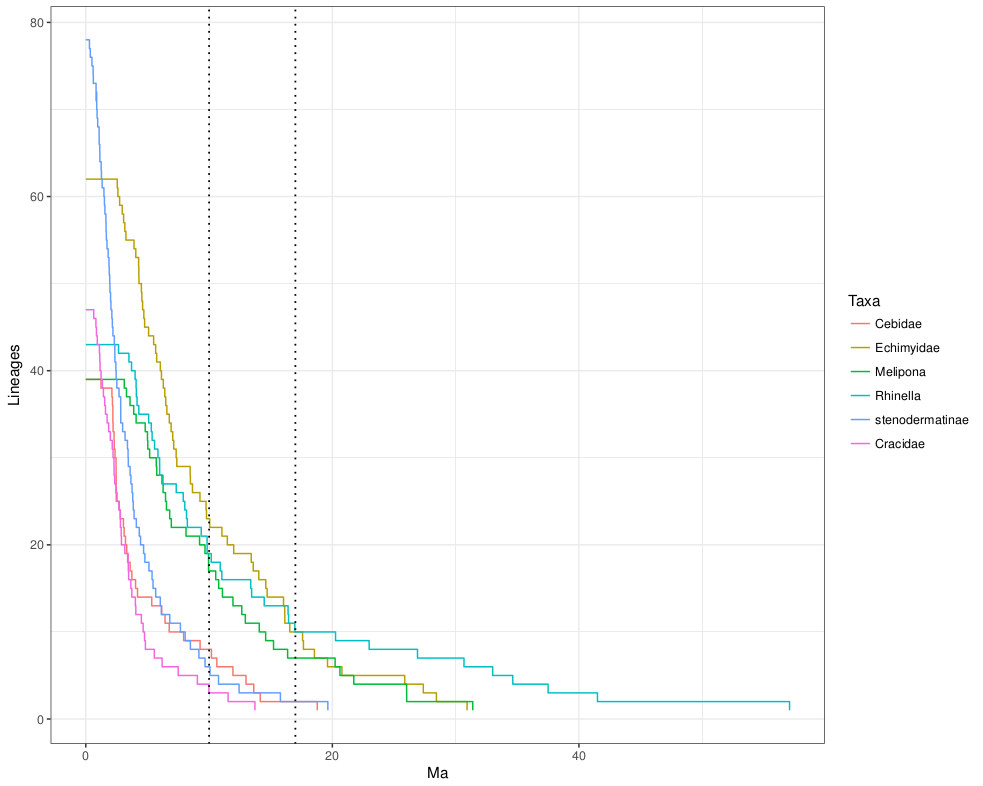
**

**Figure S3.**

**
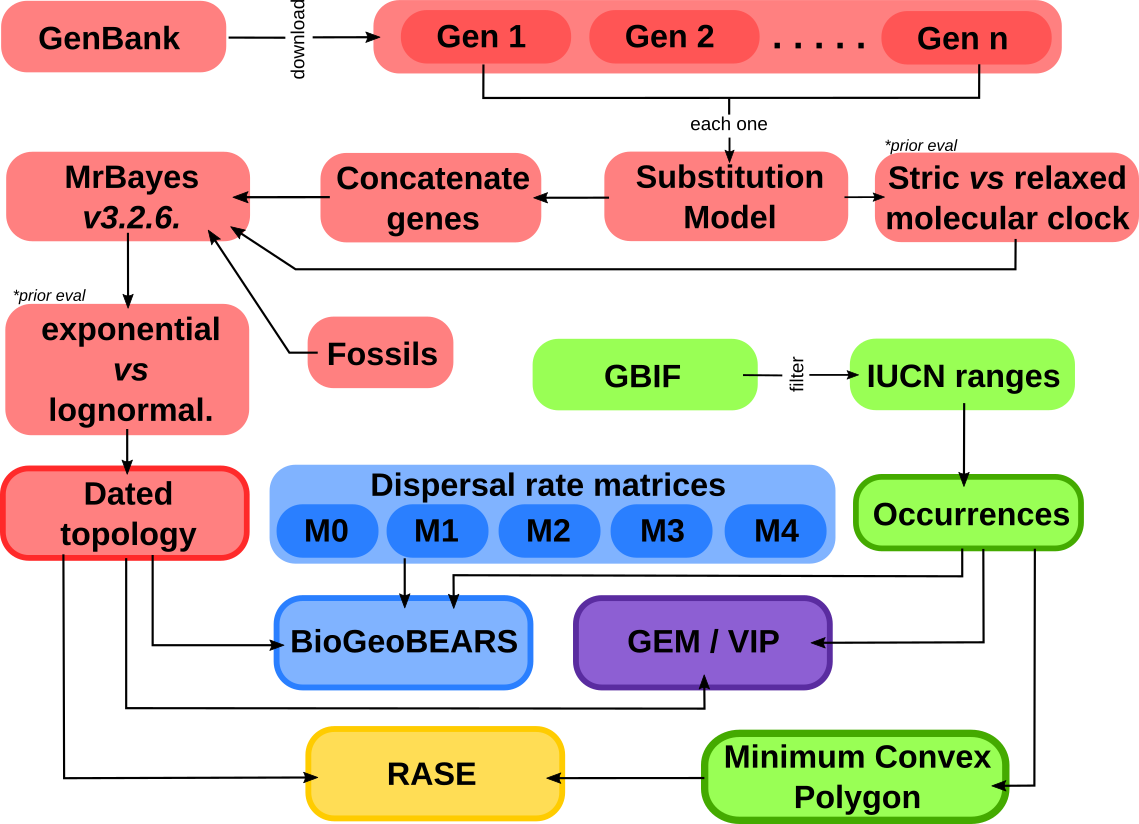
**

**Supplementary references**

Antoine PO, Abello MA, Adnet S, Altamirano Sierra AJ, Baby P, Billet G, Boivin M, Calderón Y, Candela A, Chabain J, Corfu F, Croft DA, Ganerød M, Jaramillo C, Klaus S, Marivaux L, Navarrete RE, Orliac MJ, Parra F, Pérez ME, Pujos F, Rage J, Ravel A, Robinet C, Roddaz M, Tejada-Lara JV, Vélez-Juarbe J, Wesselingh FP, Salas-Gismondi R. 2016. A 60-million-year Cenozoic history of western Amazonian ecosystems in Contamana, eastern Peru. *Gondwana Research* 31:30–59.

Báez AM, Gasparini ZB. 1979. The South American herpetofauna: an evaluation of the fossil record. In: Duellman WE. ed. *The SouthAmerican herpetofauna: its origin, evolution and dispersal*. Museum of Natural History, University of Kansas: 29–55.

Brodkorb P. 1958. Birds from the Middle Pliocene of McKay, Oregon. *The Condor* 60:252–255.

Brodkorb P. 1964. Catalogue of fossil birds: Part 2 (Anseri-formes through Galliformes). *Bulletin of the Florida State Museum* 8:195–335.

Czaplewski NJ, Takai M, Naeher TM, Shigehara N, Setoguchi T. 2003. Additional bats from the middle Miocene La Venta fauna of Colombia. *Revista de la Academia Colombiana de Ciecias exactas, físicas y naturales* 27:263–282.

Engel MS. 1999. The first fossil Euglossa and phylogeny of the orchid bees (Hymenoptera: Apidae; Euglossini. *American Museum Novitates* 3272:1–14.

Engel MS. 2006. A giant honey bee from the middle Miocene of Japan (Hymenoptera: Apidae). *American Museum Novitates* 3504:1–12.

Eastman CR. 1900. New fossil bird and fish remains from the MiddleEocene of Wyoming.*Geological Magazine* 7: 54–58.

Eastman CR. 1905. Fossil avian remains from Armissan. *Memoirs of the Carnegie Museum* 2:131–138.

Hershkovitz P. 1970. Notes on Tertiary Platyrrhine monkeys and description of a new genus from the Late Miocene of Colombia. *Folia Primatologica* 12:1–37.

Hoffstetter MR. 1969. Un primate de l’Oligoce`ne in-fe ́rieur sudamericain:Branisella bolivianagen. et sp.nov. *Comptes Rendus de l’Académie des Sciences de Paris Série D* 269:434–437.

Kay RF. 1994. "Giant" tamarin from the Miocene of Colombia. *American Journal of Physical Anthropology* 95:333–353.

Mancina CA, García-Rivera L. 2005. New genus and species of fossil bat (Chiroptera: Phyllostomidae) from Cuba. *Caribbean Journal of Science* 41:22–27.

Marivaux L, Adnet S, Altamirano‐Sierra AJ, Pujos F, Ramdarshan A, Salas‐Gismondi R, Tejada-Lara JV, Antoine PO. 2016. Dental remains of cebid platyrrhines from the earliest late Miocene of Western Amazonia, Peru: Macroevolutionary implications on the extant capuchin and marmoset lineages. *American Journal of Physical Anthropology* 161:478–493.

Mayr G, Poschmann M, Wuttke M. 2006. A nearly complete skeleton of the fossil galliform bird Palaeortyx from the late Oligocene of Germany. *Acta Ornithologica* 41:129–135.

Pérez CM, Gómez RO, Báez AM. 2014. Intraspecific morphological variation and its implications in the taxonomic status of ‘Bufo pisanoi,’ a Pliocene anuran from eastern Argentina. *Journal of Vertebrate Paleontology* 34:767–773.

Pérez MA, Arnal M, Boivin M, Vucetich MG, Candela A, Busker F, Mamani Quispe B. 2018. New caviomorph rodents from the late Oligocene of Salla, Bolivia: taxonomic, chronological, and biogeographic implications for the Deseadan faunas of South America. *Journal of Systematic Palaeontology* 17:821–847.

Poinar GO, Danforth BN. 2006. A fossil bee from Early Cretaceous Burmese amber. *Science* 314:614.

Rosenberger AL. 1981. A mandible ofBranisella bolivi-ana(Platyrrhini, Primates) from the Oligocene of South America. *International Journal of Primatology* 2:1–7.

Shotwell JA. 1956. Hemphillian mammalian assemblage from northeastern Oregon. *Geological Society America Bulletin* 67:717–738.

Stirton RA, Savage DE. 1950. A new Monkey from the La Venta Miocene of Colombia. *Compilación de los estudios geológicos oficiales en Colombia* 8:345–356.

Stirton RA. 1951. Ceboid monkeys from the Miocene of Colombia. *University of California Publications in Geological Sciences* 28:315–356.

Storch G, Engesser B, Wuttke M. 1996. Oldest fossil record of gliding in rodents. *Nature* 379:439–441.

Suárez W, Díaz-Franco S. 2003. A new fossil bat (Chiroptera: Phyllostomatidae) from a Quaternary cave deposit in Cuba. *Caribbean Journal of Science* 39:371–377.

Takai M, Anaya F, Shigehara N, Setoguchi T. 2000. New fossil materials of the earliest new world monkey, Branisella boliviana, and the problem of platyrrhine origins. *American Journal of Physical Anthropology* 111: 263–81.

Tambussi CP. 1998. Nuevo Anatidae (Aves: Anseriformes) del Plioceno de la región pampeana, Argentina. *Bolletí de la Societat d'Història Natural de les Balears* 41:19–25.

Verzi DH, Vucetich MG, Montalvo CI. 1995. Un nuevo Eumysopinae (Rodentia, Echimyidae) de Mioceno tardío de la Provincia de La Pampa y consideraciones sobre la historia de la subfamilia. *Ameghiniana* 32:191–195.

Vucetich MG, Vieytes EC, Pérez ME, Carlini AA. 2010. The rodents from La Cantera and the early evolution of caviomorphs in South America. In: Madden RH, Carlini AA, Vucetich MG, Kay RF. ed. *The Paleontology of Gran Barranca: Evolution and Environmental Change through the Middle Cenozoic of Patagonia.* Cambridge: Cambridge University Press: 189–201.

Weidig I. 2010. New Birds from the Lower Eocene Green River Formation, North America. *Records of the Australian Museum* 62:29–44.

Wetmore A. 1943. Fossil birds from the Tertiary deposits of Florida. *Proceedings of the New England Zoological Club* 22:59–68.

White TE. 1942. The Lower Miocene mammal fauna of Florida. *Bulletin of the Museum of Comparative Zoology* 92:1–49.

Wille A, Chandler LC. 1964. A new stingless bee from the Tertiary amber of the Dominican Republic (Hymenoptera; Meliponini). *Revista de Biología Tropical* 12:187–195.
